# PD-L1 Improves Motor Function and Alleviates Neuropathic Pain in Male Mice After Spinal Cord Injury by Inhibiting MAPK Pathway

**DOI:** 10.3389/fimmu.2021.670646

**Published:** 2021-04-15

**Authors:** Fanqi Kong, Kaiqiang Sun, Jian Zhu, Fudong Li, Feng Lin, Xiaofei Sun, Xi Luo, Changzhen Ren, Lantao Lu, ShuJie Zhao, Jingchuan Sun, Yuan Wang, Jiangang Shi

**Affiliations:** ^1^ Department of Orthopedic Surgery, Spine Center, Changzheng Hospital, Naval Medical University, Shanghai, China; ^2^ Department of Orthopedics, The First Affiliated Hospital of Nanjing Medical University, Nanjing, China; ^3^ Department of Cardiology, Changzheng Hospital, Naval Medical University, Shanghai, China

**Keywords:** PD-L1, spinal cord injury, neuropathic pain, macrophages/microglial, polarization, ERK1/2, p38

## Abstract

**Background:**

Traumatic spinal cord injury (SCI) causes severe motor dysfunction and persistent central neuropathic pain (Nep), which has not yet been effectively cured. Programmed cell death ligand-1 (PD-L1) is typically produced by cancer cells and contributes to the immune-suppressive in tumor microenvironment. However, the role of PD-L1 in regulating inflammatory response and Nep after SCI remains unclear. A growing amount of researches have begun to investigate the effect of PD-L1 on macrophages and microglia in recent years. Considering the pivotal role of macrophages/microglia in the inflammatory response after SCI, we proposed the hypothesis that PD-L1 improved the recovery of locomotor and sensory functions after SCI through regulating macrophages and microglia.

**Methods:**

The mice SCI model was established to determine the changes in expression patterns of PD-L1. Meanwhile, we constructed PD-L1 knockout mice to observe differences in functional recovery and phenotypes of macrophages/microglia post-SCI.

**Results:**

In present study, PD-L1 was significantly upregulated after SCI and highly expressed on macrophages/microglia at the injury epicenter. PD-L1 knockout (KO) mice showed worse locomotor recovery and more serious pathological pain compared with wild-type (WT) mice. Furthermore, deletion of PD-L1 significantly increased the polarization of M1-like macrophages/microglia. Mechanistic analysis revealed that PD-L1 may improve functional outcomes following SCI by inhibiting phosphorylation of p38 and ERK1/2.

**Conclusions:**

Our observations implicate the involvement of PD-L1 in recovery of SCI and provide a new treatment strategy for the prevention and treatment of this traumatic condition.

## Introduction

Traumatic spinal cord injury (SCI) with high morbidity and mortality is still a devastating problem worldwide, resulting in permanent motor and sensory dysfunction (1). Globally, the annual incidence of SCI has been estimated to range between 15 and 40 cases per million, with falls and road injuries being the leading causes (2). More than two-thirds of individuals with SCI suffer from chronic neuropathic pain (Nep) (3). Although there has been considerable progress in understanding the mechanism of pain process, Nep remains an area of significant unmet medical need because of limited effective targets (4, 5).

Previous studies have argued that Nep is attributed to a dysregulation and hyperexcitability of primary afferent pathways (6). However, recent evidence suggests that neuroinflammation may also be involved in Nep after SCI (7). The activation of microglia is thought to contribute to the maintenance of Nep post-SCI (8). In addition, due to the destruction of the blood‐spinal cord barrier (BSCB), a large number of blood-derived macrophages accumulate in the lesion epicenter and lead to an intensive inflammatory response (9). Infiltrating macrophages and microglia are highly sensitive to microenvironmental signals, in response to which they would undergo either pro-inflammatory (M1-like) or anti-inflammatory (M2-like) polarization (10). The imbalance of M1-like / M2-like aggravates the secondary injury and impedes the recovery of sensory and related motor function. In response to peripheral nerve injury, macrophages contribute to the pathogenesis of Nep through the release of pro-inflammatory mediators and modulation of ion channels (11, 12). Understanding the contribution of macrophages and microglia to SCI‐induced Nep is important to direct the search for novel therapies.

Programmed death ligand 1 (PD-L1; also called B7-H1 or CD274), is a B7 family member and the first functionally characterized ligand of programmed death receptor 1(PD-1) (13). Extensive works have mainly focused on the impact of PD-1/PD-L1 pathway on T-lymphocyte tolerance in tumor immune escape (14, 15). In recent years, attention has become focused on the relationship of PD-L1 to macrophage activation and polarization (16, 17). Yao et al. also reported that deficiency of PD1 promoted M1 phenotype of macrophages/microglia after SCI (18). Meanwhile, the function of PD-L1 outside the treatment of tumors is gradually discovered, including murine lupus (19), colitis (20), collagen-induced arthritis (21) and brain injury (22). Gang et al. also found that PD-L1 could inhibit acute and chronic pain after nerve injury (23).

In the present study, we assessed the expression and function of PD-L1 in spinal cord after SCI, and found that increased expression of PD-L1 potently improved functional recovery and alleviated Nep after SCI. Specifically, we focused on the effects of PD-L1 on macrophages/microglia polarization and MAPK signal pathway at the level of the lesion.

## Methods

### Animals and Spinal Cord Injury Model

PD-L1 knockout (KO) male mice (C57BL/6 background) were purchased from Shanghai Model Organisms Center (Shanghai, China), and PD-L1 Wild-type (WT) male mice with identical genetic backgrounds were used as the controls. Genotyping was confirmed by PCR of tail DNA samples (**Supplementary Figure 1**). All animal experiments were performed in accordance with guidelines established by the Animal Committee of Naval Medical University. Male C57BL/6 mice, 8-10 weeks old, were used to generate the SCI model as previously described (24). Briefly, under isoflurane gas anesthetic and sterile conditions, a T10 laminectomy was performed to expose the underlying thoracic segment of spinal cord. Then, a weight-drop device (68097, RWD, CA, USA) was used to create a moderate injury by dropping a 5g weight rod from a height of 5cm onto the exposed dorsal surface of the spinal cord. The overlying muscles and skin were then closed separately. Sham mice were subjected to laminectomy without SCI. One hour after SCI surgery, PD-L1 antibody (100ng in 20μL normal saline, NS) (124301, Biolegend, California, USA) and PD-L1 protein (100ng in 20μL NS) (ab216261, Abcam, Cambridge, UK) were administered by lumbar puncture injection with a 30G sterilized Hamilton syringe in approximately 3 mm rostral to the lesion epicenter, and then injected once a week until sacrifice. The bladders of mice were manually voided two times per day until a reflex bladder was established. The overall study protocol showed more detail in the **Supplementary Figure 2**.

### Basso Mouse Scale (BMS) Behavioral Analysis

Basso Mouse Scale was designed to evaluate the recovery of motor function of hindlimb following SCI. Mice were recorded in an open field and two independent raters were blinded to the groups of treatment. Multiple aspects of locomotion were accessed including ankle movements, trunk position and stability, stepping pattern and coordination, paw placement and tail position, with a minimum score of 0 (no ankle movement) to a maximum score of 9 (complete functional recovery). The test was carried out pre-operatively and on post-operative days 1, 3, 7 and then weekly until day 28.

### The Louisville Swim Scale

To obtain information about locomotor performance in the absence of cutaneous and proprioceptive input from the limbs to the spinal cord, the Louisville Swim Score (LSS) was used as previously described (24). In brief, mice were trained for several days, until they had learned to swim from one end of a water-filled glass tank to a visible escape platform at the opposite end. Swimming performance was evaluated including forelimb dependency, hindlimb alternation, sagittal and coronal balance, tail position and body angle, with a score of 0 to 15. Each mouse was tested twice in a double-blind manner. The test was administered pre-operatively, and 3, 7, 14, 21, 28 and 35 days post-operatively, respectively.

### Mechanical Allodynia and Thermal Hyperalgesia

The Von Frey filament test (Aesthesio, RWD) was used to assessed the mechanical allodynia of mice after SCI and investigators were blinded to the genotype and treatment of the groups. Before behavioral testing, individual mouse was placed in glass chamber on an elevated wire mesh grid and acclimated to the testing cage for 1h. Then, von Frey filaments (range from 0.04 g to 2.0 g) were applied to the lateral plantar surface of each hind paw to measure the mechanical threshold. Mechanical nociceptive thresholds were determined as the lowest filament force that resulted in a positive withdrawal response in greater than 50% of the trials. Both left and right hind limbs were tested 3 times with 5 min intervals and averaged.

After 1 hour of acclimation to the test chamber, thermal nociceptive thresholds were evaluated by measuring the latency of paw withdrawal (PWL) in response to a radiant heat source (II TC Life Science Model 390, USA). The device was adjusted to obtain a baseline withdrawal latency (10 s) in naive animals, and the cut-off latency was set at 15 sec to avoid tissue damage. 40 w infrared heat source was located below the middle plantar area of the hind paws through the heat-conducting glass floor. We wrote down the time taken from the commence of stimulus to the paw withdrawal. Both left and right hind limbs were tested 3 times with 5 min intervals, the mean value was recorded as the nociceptive latency. To minimize the stress to the animals, thermal nociceptive thresholds was tested 1 day after Frey filament test.

### Electrophysiology

Motor-evoked potentials (MEPs) of mice were recorded by electromyography to analyzed functional recovery at day 28 post-injury. Mice were firstly anesthetized with halothane (3% induction, 1–1.5% maintenance) in oxygen (0.4 L/min) and nitrogen (0.6 L/min). After that, a stimulation electrode was placed dorsally on the rostral ends of the surgically exposed spinal cord, the recording electrode was placed in the biceps femoris flexor cruris, the reference electrode was placed over the distal tendon of the muscle in the hindlimb, and the ground electrode was placed subcutaneously. Electrical stimulation (0.5 mA, 0.5 ms, 1 Hz) was applied and peak-to-peak amplitude of MEPs was used to measured nerve conduction function.

### Magnetic Resonance Imaging (MRI)

Mouse MRI experiments were performed on a small animal MRI system (Bruker BioSpec 7T/20 USR, Germany). The sequence protocol included T2-weighted, 256×256 matrix, slice thickness 1 mm, intersection gap 1 mm, echo time (TE)/repetition time (TR) 27/3000 ms, RARE factor 16, and flip angle 90°. T2-weighted images were acquired in the sagittal and axial planes by the ParaVision 6.0.1 (Bruker BioSpec, Germany). The area of the lesioned spinal cord containing hyperintense signal was first manually traced by a blinded observer. A computer-aided software (FireVoxel; CAI2R, New York University, NY) was used for axial images to assess and compare the evolution of hyperintense signal and lesion volume obtained by adding the individual slice areas and multiplying by 1.0 mm slice plus gap thickness. For quantitative analysis, the results were calculated by the intensity ratio for the signal of spinal cord lesion to normal cord far from injury area.

### Western Blotting

Mouse spinal cord tissues (epicenter ± 0.3 cm) and DRG samples were collected and homogenized with 10 strokes of a homogenizer at 4°C in protein extraction buffer. Subsequently, protein concentrations were determined by the BCA method, and equal amounts of proteins were separated by 10% SDS-PAGE gel electrophoresis and transferred onto polyvinylidene fluoride (PVDF) membranes. After blocking with 5% skimmed milk or 5% Bovine Serum Albumin (BSA) for 2h at room temperature, the membranes were incubated overnight at 4°C with the following corresponding primary antibodies: anti-PD-L1 (1:1000, ab213480, abcam), anti-TRPV1 (1:1000, 508564, ZEN BIO, Chengdu, China), anti-iNOS (1:1000, ab178945, abcam), anti-Arg1 (1:1000, 16001-1-AP, proteintech, Wuhan, China), anti-β-actin (1:1000, ab123946, abcam), anti-ERK (1:1000, 4695, CST, Massachusetts, USA), anti-pERK1/2 (1:1000, 4370, CST), anti-p38 (1:1000, 8690, CST), anti-pP38 (1:1000, 4511, CST), anti-JNK (1:1000, 9252, CST), anti-pJNK (1:1000, 9255, CST). After washing and incubating with species-matched secondary antibodies (1:5000) at RT for 2 h, immunoreactive bands were visualized using ECL reagents (PE0010, Solarbio, Beijing, China) and the density of protein was quantified by the ImageJ (National Institutes of Health, Bethesda, MD, USA)

### Quantitative Real-Time PCR (qRT-PCR) Analysis

Total RNA of spinal cord (epicenter ± 0.3 cm) was isolated using the Trizol reagent (Takara, Dalian, China) and subsequently transcribed into cDNA by HiScript II Q RT SuperMix for qPCR (R122-01, Vazyme, Nanjing, China). Next, qPCR was performed using AceQ qPCR SYBR Green Master Mix (Q111-02, Vazyme) in a 7500 real-time PCR system (Applied Biosystems, Inc., USA). The reaction conditions were designed as follows: 40 cycles of denaturation at 95°C for 10 s and amplification at 60°C for 30 s. The primer sequences are listed in **Supplementary Table 1**. The mRNA levels of target genes were normalized to GAPDH expression. Relative quantification of gene expression was performed by the 2^−△△^CT method.

### Histology and Immunofluorescence

At different time points, mice were transcardially perfused with 0.9% saline followed by 4% paraformaldehyde, the spinal lesion segments and DRG (L4 and L5) were removed and fixed overnight in 4% paraformaldehyde. After dehydration in graded sucrose solutions (15–30%), tissues were immersed in OCT and cut into 10-μm-thick serial sections for subsequent experiments. To ensure the same organizational level, the tissue sections were numbered and the same serial number was used, three sequential sections of spinal cord and DRG tissue at 50 µm intervals per mouse were selected for staining and quantification. For immunofluorescence staining, non-specific binding was blocked with 10% BSA for 30 min. Then histological sections were incubated overnight at 4 °C with the following primary antibodies: anti-NF200 (1:200, ab4680, abcam), anti-GFAP (1:500, ab7260, abcam), anti-TRPV1 (1:200, 508564, ZEN BIO), anti-iNOS (1:200, ab15323, abcam), anti-ARG1 (1:200, 16001-1-AP, Proteintech), anti-IBA-1(1:200, MA5-27726, Thermo Fisher), anti-CD11b (1:200, ab8878, abcam), anti-PD-L1 (1:200, ab213480, abcam), anti-Neuron (1:300, ab177487, abcam), anti-CD31(1:200, GB11063-2, Servicebio), anti-OLIG2 (1:200, ab109186, abcam), followed by secondary antibodies for 2 h at room temperature. The nuclei were then stained with DAPI, and images were obtained using a confocal microscope (LSM710, Zeiss, Heidenheim, Germany). Immunoreactive signals were quantified by measuring the intensity of fluorescent signal or the number of immunoreactive cells. For the relative quantification of immunofluorescence staining, fluorescent signal intensity was measured in three random visual fields per section using ZEN software (version ZEN 2012 SP1, Zeiss) and mean values were calculated.

### TUNEL Assay

Apoptotic cells were detected with the TUNEL Apoptosis Assay Kit (T2190-50 T, Servicebio) according to the manufacturer’s instructions. The nuclei were stained with DAPI, and images were acquired by a confocal microscope (LSM710, Zeiss, Heidenheim, Germany).

### Isolation of CD11b+ Macrophages/Microglia

Mice were humanely sacrificed by CO2 and perfused with ice-cold PBS. The spinal columns were dissected and the spinal cord isolated quickly from the vertebrae. Single cell suspensions were prepared using the MACS Neural Dissociation Kit (130-107-677, Miltenyi, Auburn, CA) according to manufacturer’s instructions. Myelin was removed and cell suspensions were incubated with CD11b microbeads (130-093-634, Miltenyi) and placed in the magnetic field of a MACS separator. The negative fraction (flow through) was collected, and the column was washed three times with MACS buffer.

### Flow Cytometry Analysis

To investigate the polarization of macrophages/microglia, single cell suspensions from spinal cord were collected and CD11b-selected cells were stained using anti-CD11b-FITC (1:200, 561688, BD), iNOS-PE (1:200, 61-5920-82, Thermo), and CD206-APC (1:200, 17–2061-82, Thermo) for 30 min on ice in the dark. Cells were analyzed by flow cytometry (FACSVerse 8, BD) and data analysis was performed using the FlowJo software (Version 7.6.1, Treestar, Ashland, OR, USA)

### Statistical Analyses

Data analysis was conducted with SPSS 18.0 statistical software (Chicago, USA), and the obtained data were presented as mean ± standard deviation (SD) values. Behavioral assays in different groups were analyzed by repeated measurement analysis of variance (ANOVA) followed by Tukey’s post hoc test. One-way ANOVA followed by the Bonferroni’s post hoc test was used for the other multiple comparisons in this study, and unpaired 2-tailed Student’s t tests were used for two-group comparisons. When p < 0.05, the differences were considered to show statistical significance.

## Results

### Spatiotemporal Patterns of PD-L1 Expression After Spinal Cord Injury

**Figure 1:**
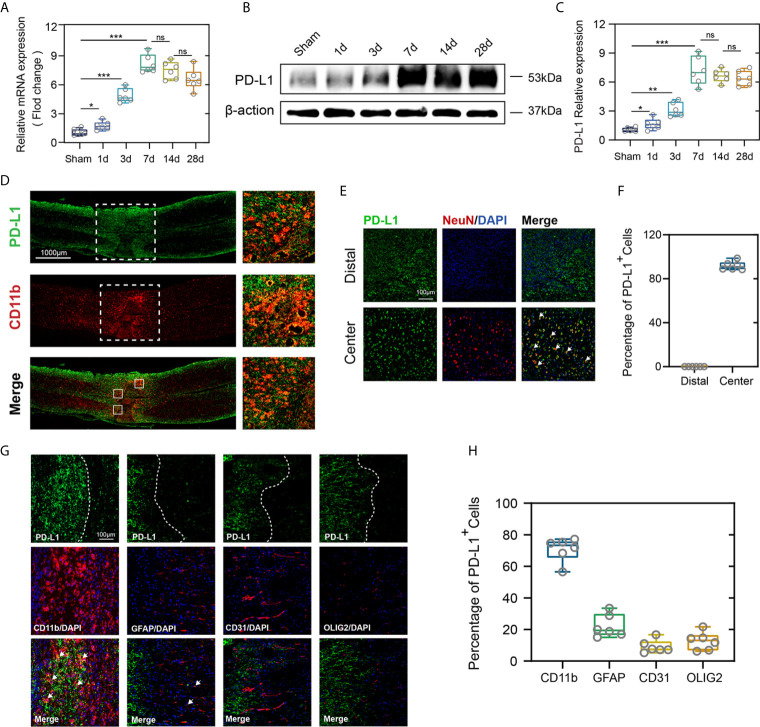
PD-L1 increased mainly in macrophages/microglia at the injury epicenter. **(A–C)** Quantitative PCR and immunoblotting of PD-L1 at days 1, 3, 7, 14, 28 after SCI or sham surgery, each lane corresponds to protein from an individual mouse. (n = 6 mice per group at each time point, values are the mean ± SD, *p < 0.05, ns indicates no significance, **P < 0.01, ***P < 0.001, one-way ANOVA). **(D)** Immunofluorescence (IF) staining of the macrophages/microglia biomarker, CD11b (red), and PD-L1 (green) in the spinal cord on day 7 post-SCI. **(E)** Representative immunofluorescence images for NeuN (red) and PD-L1 (green) expression in distal and center of SCI. **(F)** Quantification of NeuN^+^PD-L1^+/^NeuN^+^ cells at day 14 post-injury. **(G)** Immunofluorescence (IF) staining of the macrophages/microglia biomarker, CD11b (red), and astrocyte biomarker, GFAP (red), and endothelial cell marker, CD31 (red), and oligodendroglia marker, Olig2 (red) and PD-L1 (green) respectively. Nuclei were counterstained with DAPI (blue). **(H)** Quantification of double-positive/single-positive cells in central lesion core at day 14 post-injury.

To demonstrate the potential role of PD-L1 in the pathophysiology of SCI, expression pattern of PD-L1 in the spinal cord (0.3 cm around injury site) was assessed by qRT-PCR and western blot in uninjured and SCI mice at different timepoints. In comparison with the sham operation group, both PD-L1 mRNA and protein levels were significantly upregulated in the SCI group, with a peak appearing on day 7 and remaining high at 14 and 28 days (**Figures 1A–C**). More importantly, the PD-L1 staining was predominantly concentrated at injury segment and co-localized with CD11b positive macrophages/microglia (**Figure 1D**). To define the cell specificity of PD-L1 expression in the spinal cord after SCI, we performed co-staining of PD-L1 with neuron, macrophages/microglia, astrocyte, endothelial cell and oligodendroglia markers at day 14 post-SCI. As shown in **Figures 1E, F**, approximately 92% of neurons (NeuN) expressed PD-L1 in the non-injured distal of spinal cord and absence of any positive staining of NeuN at the injury epicenter. Meanwhile, PD-L1 immunoreactivity was co-stained mainly with macrophages/microglial marker CD11b, to a lesser extent, with astrocytic marker GFAP, endothelial cell marker CD31 and oligodendroglia marker Olig2 in the damaged center after SCI (arrows indicated the positive cells, **Figures 1G, H**).

### PD-L1 Promoted Functional Behavioral Recovery and Reduced Cellular Damage After SCI

**Figure 2:**
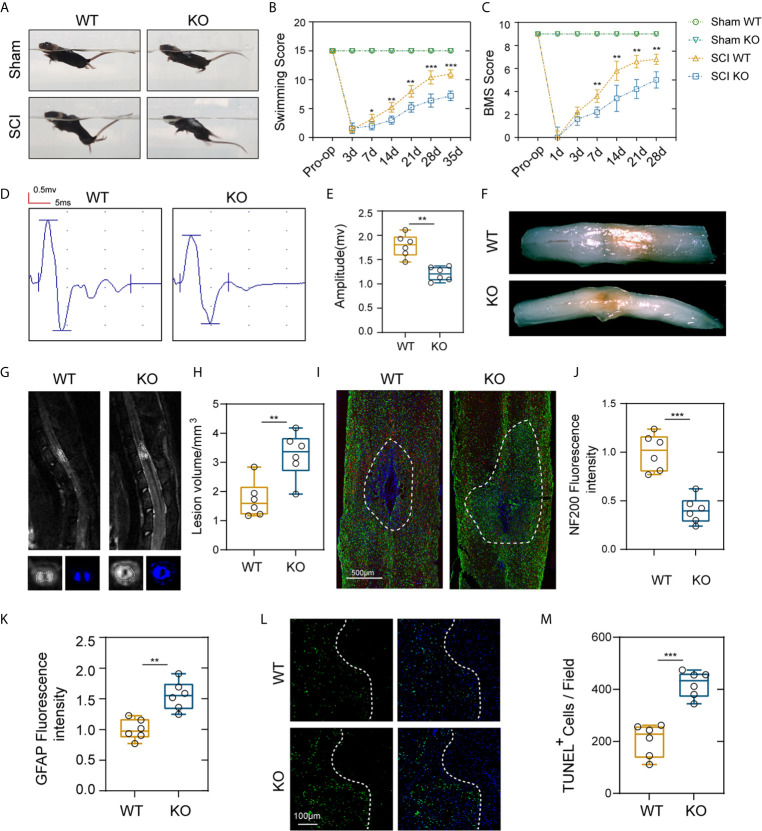
PD-L1 deficiency suppressed motor function recovery and promoted spinal cord damage after SCI. **(A, B)** Representative images of swimming test after SCI or sham surgery, and statistical analysis of the Louisville Swim Scale in the PD-L1 WT and KO groups over a 35-days period (n = 5 mice per group at each time point, values are the mean ± SD, *p < 0.05, **p < 0.01, repeated measurement analysis of variance). **(C)** Statistical analysis of the Basso Mouse Scale (BMS) in the PD-L1 WT and KO groups over a 28-days period (n = 5 mice per group at each time point, values are the mean ± SD, **p < 0.01, repeated measurement analysis of variance). **(D, E)** Electrophysiological analyses of PD-L1 WT and KO mice at day 28 post-SCI (n = 6 mice per group, values are the mean ± SD, *p < 0.05, **p < 0.01, two-tailed Student’s t tests). **(F)** Gross morphology of the injured spinal cords in PD-L1 WT and KO mice at day 28 post-SCI. **(G, H)** Representative images of MRI in PD-L1 WT and KO mice at day 28 post-SCI (n = 6 mice per group, values are the mean ± SD, **p < 0.01, two-tailed Student’s t tests). **(I)** Representative images of GFAP (green) and NF200 (red) in the lesion sites of the PD-L1 WT and KO groups at day 28 post injury, nuclei were counterstained with DAPI (blue); the dashed lines indicate the boundary of the damaged area (n = 6 mice per group, scale bar = 500 μm). **(J, K)** Quantification of the NF200 and GFAP intensity (n = 6 mice per group, values are the mean ± SD, **p < 0.01, ***p < 0.001, two-tailed Student’s t tests). **(L, M)** Representative images showing TUNEL and DAPI co-staining of spinal cord sections of the PD-L1 KO and WT mice at day 28 post-SCI (n = 6 mice per group, values are the mean ± SD, ***p < 0.001, two-tailed Student’s t tests, scale bar = 100 μm).

To test whether PD-L1 expression is associated with locomotor recovery after SCI, we constructed PD-L1 knockout mice in C57BL/6J background. The motor function recovery of the PD-L1 wild-type (WT) and knockout (KO) mice after SCI was estimated by visual observation according to the Louisville Swim Scale and Basso Mouse Scale (BMS) system. As indicated in **Figures 2A–C**, no apparent difference was demonstrated in the swimming test and BMS scores between the two groups of mice in sham groups. By contrast, the Louisville Swim Scale score for the PD-L1 KO mice was significantly lower than that of the PD-L1 WT mice beginning on day 7 after SCI (**Figures 2A, B**). The BMS score also indicated that PD-L1 KO mice drastically attenuated motor recovery after SCI compared with WT mice (**Figure 2C**). To further objectively evaluate the recovery of motor function, electrophysiological analyses were applied at day 28 post-injury. MEP amplitudes were lower in the PD-L1 KO mice than in the WT group, indicating a worse electrophysiological recovery (**Figures 2D, E**). Four weeks following SCI, as indicated by the gross morphology of the injured spinal cords, the traumatic lesion areas (brown colored region) of PD-L1 KO mice were notably larger than that in WT group (**Figure 2F**). Magnetic resonance imaging (MRI) was also used to calculate lesion volume, hemorrhage and edema were recognized by hyperintense signal in T2 weighted images. Representative images of MRI confirmed that knockout of PD-L1 significantly increased the lesion area compared with the WT group (**Figures 2G, H**). Neurofilament is a surrogate marker of axonal degeneration in central nervous system (CNS) diseases (25). In addition, the formation of dense glial scars impedes neurofilament regeneration and remyelination (26, 27). To further elucidate the anatomical basis of the observed phenomenon, immunostaining was performed for 200kDa subunit of neurofilament (NF200) and glial fibrillary acidic protein (GFAP). As shown in **Figures 2I–K**, the fluorescence intensity of NF200 was lower and fluorescence intensity of GFAP was stronger in lesion site of PD-L1 KO group compared with WT mice. The TUNEL assay also confirmed that the percentage of TUNEL-positive cells was higher in the lesion core of PD-L1 KO group than in WT group (**Figures 2L, M**). Combined with the results above, we reasoned that PD-L1 could alleviate motor dysfunction and cellular damage after SCI.

### Genetic Deletion of PD-L1 Promotes SCI-Induced Nep

**Figure 3:**
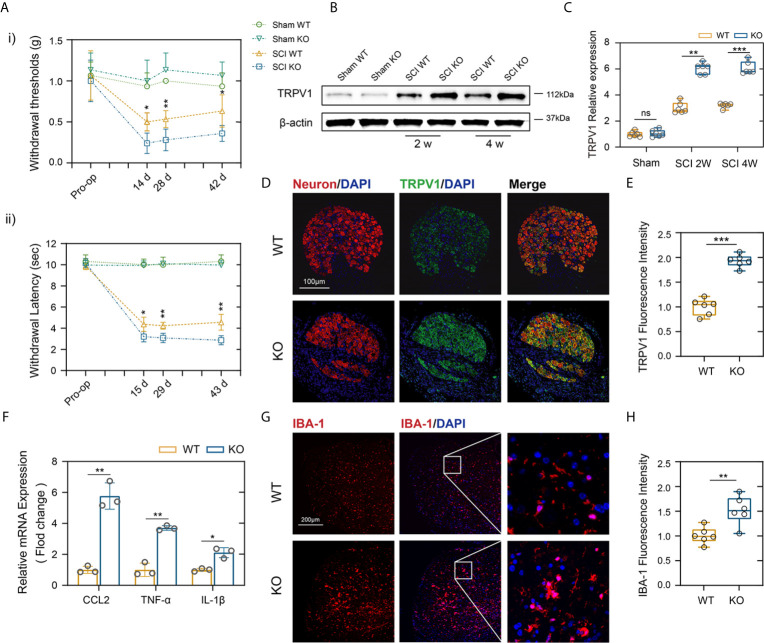
PD-L1 ablation promoted Nep after SCI. **(Ai, ii)** Statistical analysis of the mechanical and thermal threshold in PD-LI WT and KO mice. (n = 5 mice per group at each time point, values are the mean ± SD, *p < 0.05, **p < 0.01, repeated measurement analysis of variance). **(B, C)** Western blot analysis of TRPV1 proteins in DRG (L4 and L5) of PD-L1 WT and KO mice post-SCI, and each lane corresponds to protein from an individual mouse (n = 6 mice per group at each time point, values are the mean ± SD, ns indicates no significance, **p < 0.01, ***p < 0.001, one-way ANOVA). **(D, E)** IF staining of the Neuron (red) and TRPV1 (green) in the DRG of the PD-L1 WT and KO mice at day 14 post-SCI. Nuclei were counterstained with DAPI (blue). (n = 6 mice per group, values are the mean ± SD, ***p < 0.001, two-tailed Student’s t tests, scale bar = 100 μm). **(F)** Quantitative PCR of CCL2, TNF-α and IL-1β in DRG of the PD-L1 WT and KO mice at day 14 post-SCI (n = 3 mice per group, values are the mean ± SD, *p < 0.05, **p < 0.01, two-tailed Student’s t tests). **(G, H)** IF staining of the IBA-1 in the dorsal horn of PD-L1 WT and KO mice at day 14 post-SCI. Nuclei were counterstained with DAPI (blue) (n = 6 mice per group, values are the mean ± SD, **p < 0.01, two-tailed Student’s t tests, scale bar = 100 μm).

About two-thirds of patients with SCI experience Nep, and major studies of Nep have evaluated the withdrawal of von Frey-stimulated hind limbs after SCI in mice (28). The same way is also applied to quantitative sensory testing in patients, which has confirmed the presence of allodynia or dysesthesia to von Frey stimulation in regions of both at and below the injury level pain (29). As indicated in **Figure 3Ai**, there were no obvious differences in mechanical threshold stimuli between the genotypes in the sham groups. In contrast, PD-L1 KO mice had a significantly lower mechanical threshold than the WT mice on days 14 following SCI, and these differences persisted to days 42, indicating more tactile hypersensitivity (**Figure 3Ai**). A large number of experiences have assessed withdrawal of the hind paw to thermal stimuli and reported that a decrease in the withdrawal threshold indicated more sensitivity to heat stimuli, or thermal hyperalgesia (30). **Figure 3Aii** demonstrated that PD-L1 KO mice showed greater thermal hyperalgesia than WT group at days 15, 29 and 43 post SCI, respectively. Some studies reported that SCI increased transient receptor potential vanilloid-1 (TRPV1), a Ca2+-permeable, nonselective cation channel that expressed in DRG and Wu et al. reported that TRPVI channels played a major role in pain-related hypersensitivity for a long time after SCI (31). Therefore, we dissected the mice lumbar DRG (L4 and L5 ganglia) from different genotypes after SCI, western blot and immunofluorescence were used to analyze the expression levels of TRPV1 protein. As shown in **Figures 3B**, **C**, the amount of TRPV1 protein (normalized to β-actin) was significantly higher in PD-L1 KO mice compared with WT mice at weeks 2 and 4 post SCI. Similar result was also demonstrated by immunofluorescence analysis of TRPV1 in mice DRG at weeks 2 following SCI (**Figures 3D, E**). Accumulating evidence indicate that activated macrophages/microglia in dorsal horn and the secretion of chemokine CCL2 and proinflammatory cytokines (IL-1β and TNF-α) play critical roles in chronic pain after SCI (32). In this present study, the mRNA expression levels of CCL2, IL-1β and TNF-α were increased in the lesion areas of PD-L1 KO mice relative to those in the PD-L1 WT mice (**Figure 3F**). Immunofluorescence results also demonstrated that fluorescence intensity of microglia activated in the dorsal horn of PD-L1 KO group was stronger than that in the WT group (**Figures 3G, H**).

### PD-L1 Attenuates M1-Like Macrophages/Microglia Activation and Promotes M2-Like Polarization After SCI

**Figure 4:**
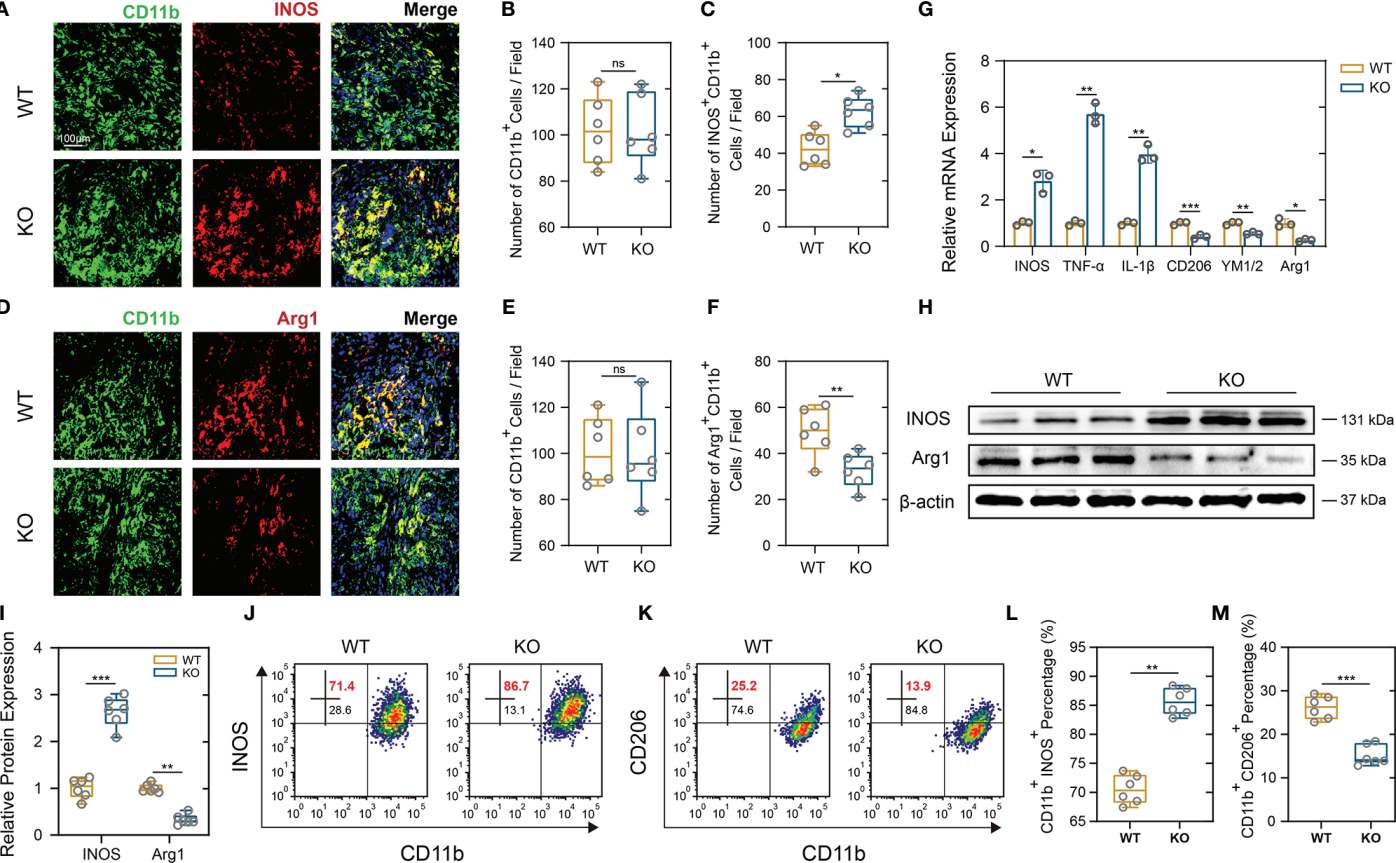
PD-L1 knockout promoted pro-inflammatory polarization of macrophages/microglia after SCI. **(A–F)** IF staining of the CD11b (green), and M1-like biomarker, iNOS (red) and M2-like biomarker, Arg1 (red), in damage area on day 14 post-SCI. Nuclei were counterstained with DAPI (blue). Bar = 100 μm. **(A, D)** The number of CD11b^+^ macrophages/microglia **(B, E)** and the number of iNOS^+^ CD11b^+^ and Arg1^+^ CD11b^+^ macrophages/microglia **(C, F)** were determined (n = 6 mice per group, values are the mean ± SD, ns indicates no significance, *p < 0.05, **p < 0.01, two-tailed Student’s t tests). **(G)** mRNA expression levels of marker genes (M1-like: iNOS, TNF-α and IL-1β, M2-like: CD206, YM1/2 and Arg1) in the damage area from PD-L1 WT and KO mice on day 14 post-SCI (n = 3 each group, *p < 0.05, **p < 0.01, ***p < 0.001, two-tailed Student’s t tests). **(H, I)** The protein expression level of iNOS and Arg2 detected by western blot in PD-L1 WT and KO mice at day-14 post SCI. (n = 6 mice per group, values are the mean ± SD, **p < 0.01, ***p < 0.001, one-way ANOVA). **(J–M)** Flow cytometry analysis of isolated CD11b^+^ cells in PD-L1 WT and KO mice at day 14 post SCI. The percentages of CD11b^+^ iNOS^+^ and CD11b^+^ CD206^+^ cells were calculated (n = 6 per group, values are the mean ± SD, **p < 0.01, ***p < 0.001, two-tailed Student’s t tests).

As a hallmark of SCI pathology, macrophage and microglia play critical roles in inflammatory response that further contribute to secondary injury (33, 34). Meanwhile, excessive macrophages/microglia infiltration and activation release proinflammatory cytokines that contribute to the persistent Nep (35). PD-L1 and its receptor PD-1 are key modulators in negatively regulating immune cell function or differentiation and have been thought to play an important role in macrophage polarization and activation (16, 17). Therefore, we further determined whether PD-L1 alters the polarization of macrophages/microglia in the SCI model. As demonstrated in **Figures 4A–F**, there were no significant differences in number of CD11b-positive cells between the two mouse groups. However, an obviously increase in the inducible nitric oxide synthase (iNOS)-positive macrophages/microglia fraction and a lower level of arginase-1 (Arg1) in the macrophages/microglia were observed in the injury areas from the PD-L1 KO mice at days 14 post SCI. The gene expression of M1-like markers (iNOS, TNF-α, IL-1β) and M2-like markers (Arg1, CD206, YM1/2) were validated using qRT-PCR. The mRNA expression levels of pro-inflammatory markers were increased whereas those of anti-inflammatory markers were decreased in the lesion site of PD-L1 KO mice compared to the WT group (**Figure 4G**). Western blot analysis also confirmed the qRT-PCR results (**Figures 4H, I**). Flow cytometry was performed in isolated CD11b-positive cells, where the ratio of M1-like macrophages (iNOS positive) in CD11b-positive cells were higher and the percentage of M2-like macrophages (CD206 positive) were lower in the PD-L1 KO group relative to WT group (**Figures 4J–M**). Consequently, those results demonstrated that PD-L1 had a significant influence on the ratio of anti-inflammatory to pro-inflammatory phenotype of macrophages/microglial post SCI and promoted M2-2like polarization.

### Intrathecal Injection of PD-L1 Inhibits the Inflammation Response and Promotes Motor Function and Sensory Recovery After SCI

**Figure 5:**
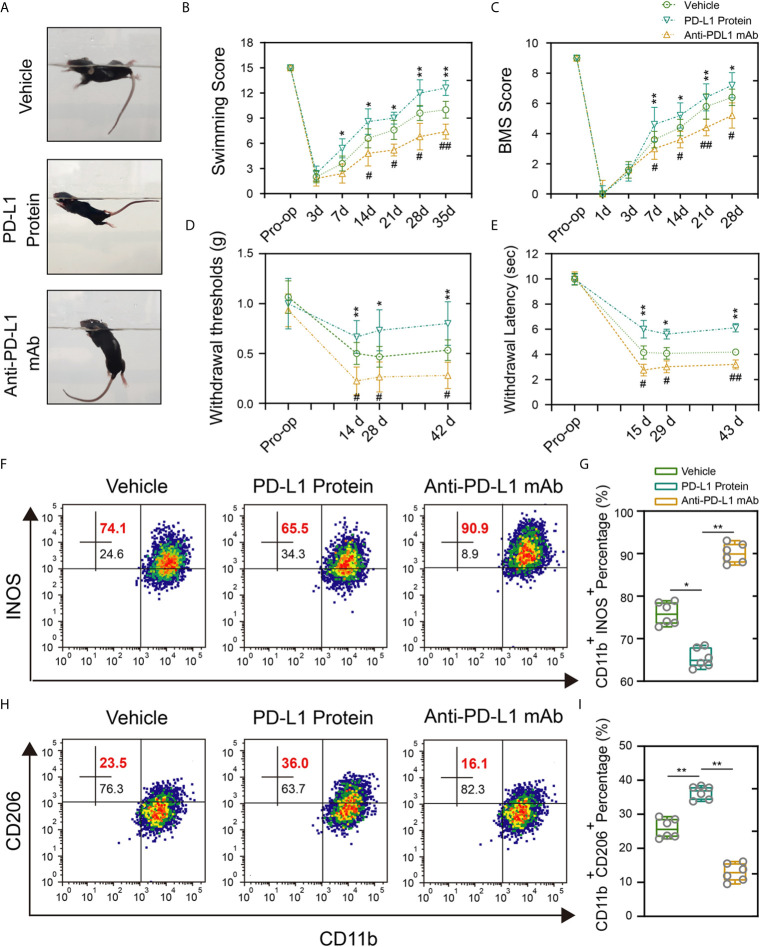
Intrathecal injection of PD-L1 promotes recovery and attenuate inflammation response. **(A–C)** Statistical analysis of the Louisville Swim Scale and BMS score of mice in different groups after SCI (n = 5 mice per group at each time point, values are the mean ± SD, *p < 0.05, **p < 0.01 vs. PD-L1 Protein, ^#^p < 0.05, ^##^p < 0.01 vs. Anti-PD-L1 mAb, repeated measurement analysis of variance). **(D, E)** Statistical analysis of the mechanical and thermal threshold of mice in different groups after SCI. (n = 5 mice per group at each time point, values are the mean ± SD, *p < 0.05, **p < 0.01 vs. PD-L1 Protein, ^#^p < 0.05, ^##^p < 0.01 vs. Anti-PD-L1 mAb, repeated measurement analysis of variance). **(F–I)** Flow cytometry analysis of CD11b^+^ iNOS^+^ and CD11b^+^ CD206^+^ cells in different groups at day 14 post-SCI (n = 6 per group, values are the mean ± SD, *p < 0.05, **p < 0.01, one-way ANOVA).

To avoid the potential developmental impact of PD-L1 knockout on macrophage s/microglial and further verify the reliability of previous results, intrathecal injection of PD-L1 protein and PD-L1 antibody was performed. The administration of PD-L1 protein significantly promoted locomotor recovery from the seventh day after SCI, as manifested by swimming test and BMS scores. Conversely, anti–PD-L1 monoclonal antibody (mAb) impaired the functional behavioral outcomes (**Figures 5A–C**). Mechanical and thermal allodynia tests revealed a significantly higher mechanical/hot threshold in PD-L1 protein group and an obviously lower in PD-L1 mAb group relative to vehicle group (**Figures 5D, E**). Meanwhile, flow cytometric analysis showed that the proportion of M1-like macrophages/microglia in the area of injury was increased, and proportion of M2-like macrophages/microglia decreased in the anti–PD-L1 mAb-treated group (**Figures 5F–I**). By contrast, PD-L1 treatment significantly reduced M1-like macrophages/microglia but increased M2-like macrophages/microglia compared with vehicle group (**Figures 5F–I**). Taken together, these findings suggested that intrathecal injection of PD-L1 can inhibit the inflammatory response and promote motor function and sensory recovery after SCI.

### PD-L1 Suppresses the SCI-induced Increase in MAPK Phosphorylation in the Injured Areas of Mice

**Figure 6:**
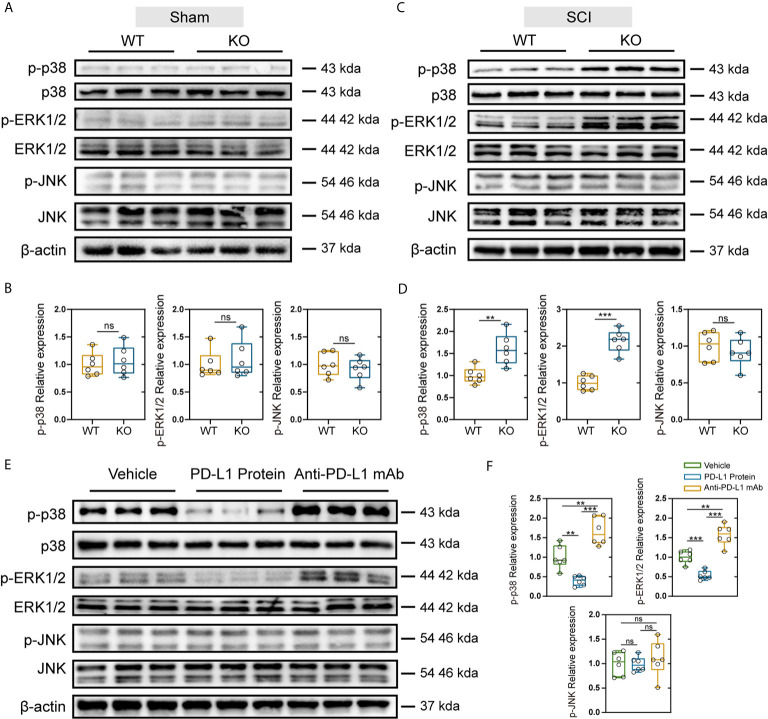
Effects of PD-L1 on phosphorylation of p38, ERK1/2, and JNK in damage area of sham group and SCI group. **(A, B)** Immunoblot images showing the effect of PD-L1 on the expression of p-p38/p38, p-ERK1/2/ERK1/2 and p-JNK/JNK in WT and KO mice without SCI. (n = 6 per group, values are the mean ± SD, ns indicates no significance, two-tailed Student’s t tests). **(C, D)** Immunoblot images showing the effect of PD-L1 on the expression of p-p38/p38, p- ERK1/2/ERK1/2 and p-JNK/JNK in WT and KO mice post-SCI. (n = 6 per group, values are the mean ± SD, ns indicates no significance, **p < 0.01, ***p < 0.001, two-tailed Student’s t tests). **(E, F)** Protein levels of p-p38/p38, p-ERK1/2/ERK1/2 and p-JNK/JNK in different groups post-SCI (n = 6 per group, values are the mean ± SD, ns indicates no significance, **p < 0.01, ***p < 0.001, one-way ANOVA).

The MAPK signaling pathway includes the activation of p38, extracellular signal regulated kinases (ERK) and c-Jun N-terminal kinase (JNK), and is associated with the induction of inflammatory factors and maintenance of Nep (36). Therefore, we further explored whether the differences observed between the PD-L1 KO and WT groups were related to the activation of MAPK signaling pathway. As indicated in **Figures 6A–D**, SCI significantly increased level of phospho-ERK1/2, phospho-p38 and phospho-JNK in injured segments at days 14 post operation. In the sham-operation groups, PD-L1 did not affect the phosphorylation of p38, ERK1/2 and JNK. However, knockout of PD-L1 obviously increased the p-p38 and p-ERK, without affecting p-JNK levels in spinal cord after SCI (**Figures 6C, D**). Moreover, phosphorylated P38 and ERK1/2 induction was alleviated by intrathecal injection of PD-L1 protein compared with vehicle group (**Figures 6E, F**). The opposite trend was observed in anti-PD-L1 mAb-treated mice with significance in p-p38 and p-ERK1/2 changes but not at the level of statistical significance in p-JNK changes (**Figures 6E, F**).

## Discussion

As a coinhibitory checkpoint molecule, PD-L1 is known to inhibit T-cell responses in tumor microenvironment (14, 15). However, few studies have been published investigating the effect of PD-L1 signaling pathway on macrophages/microglia. Besides oncology researches, the role of PD-L1 in other fields is gradually being discovered, but the effect of PD-L1 in SCI is seldom investigated (37). The present study is the first report to describe the protective effect of PD-L1 on the recovery of SCI and present a preliminary exploration of possible mechanisms through macrophages/microglia in male mice.

Neuroinflammation is a well-controlled physiological response evoked by injury and infection that promote tissue repair, regeneration and wound healing (38). Nevertheless, if the resolution of neuroinflammation is disturbed, the chronic inflammatory state will result in death of neurons and persistent Nep (39). Key players of Nep in spinal cord are macrophages, microglia, astrocytes, cytokines and chemokines. In the present study, based on the results of Western blots and qRT-PCR, the expression of PD-L1 was elevated in the damage area, and then we identified the spatiotemporal characteristics and cell types in which PD-L1 positive cells accumulated after SCI. In the non-injured area, PD-L1 was highly expressed in neurons, but in the injured center, PDL1 was mainly expressed on macrophages/microglia. It is well-accepted that macrophages/microglia can play a double-edged role in the SCI microenvironment including pro-inflammatory (M1-like) phenotype and anti-inflammatory (M2-like) phenotype (10). In the injured spinal cord, M1-like macrophages/microglia have a detrimental effect, whereas M2-like macrophages/microglia could promote regeneration of neurons and axons and ameliorate local inflammation response (10). Following SCI, most macrophages/microglial are polarized into M1-like phenotype and only a transient and small number are M2-like (40). Chronic and excessively M1-like activation releases high-level proinflammatory cytokines and contributes to the chronic inflammatory response and Nep. Our data in mice SCI model indicated that increased PD-L1 expression after SCI promoted M2-like polarization, and improved motor function recovery and Nep.

Sex difference in pain has been widely investigated, with various reviews concluding that males and females not only perceive pain differently, they also respond to analgesics differently under certain conditions (41). Females tend to be more sensitive to nociceptive stimuli in different animal models of pain. Studies have demonstrated that differences in the immune system may be related to the different observed responses of females and males to pain. Microglia in the spinal cord is utilized to mediate pain in male mice, whereas females preferentially use T cells in a similar manner (42). Considering that our study focused on the role of macrophages and microglia in Nep after SCI, only male mice were used to exclude the influence of gender on the experimental results. Nevertheless, whether PD-L1 affects pathological pain in female mice through T-cells will be further studied.

Mitogen-activated protein kinases (MAPKs) belong to a family of serine-threonine protein kinases, including ERK1/2, p38, and JNK, and are involving in upregulation of proinflammatory cytokines and Nep (43, 44). Zhang et al. reported that MAPK signaling pathway was the most significantly activated after SCI by performing bioinformatic analysis (45). Takeura et al. also confirmed that the increased phosphorylation of p38 and ERK1/2 in macrophages/microglia was associated with Nep after chronic progressive compression of the spinal cord (46). In addition, intrathecal injection of the selective inhibitors of MAPK pathways produced analgesic activities in rodent models of inflammatory, neuropathic and cancer pain (47). In the present study, we found that PD-L1 ablation or inhibition obviously increased the phosphorylation of p38 and ERK1/2, indicating that PD-L1 showed protective effects in mice SCI model by inhibiting the MAPK signaling pathway.

Although we evaluated recovery of SCI using PD-L1 KO mice, the indirect effect of other cells could not be fully excluded. Neuronal hyperexcitability and increased spontaneous activity in spinal cord and DRG are also related to Nep after SCI. Given the high expression of PD-L1 in neurons, further experiments using conditional KO mice with a higher specificity for macrophages/microglia are required to determinate the exact role of PD-L1 in SCI. In addition, as the important receptor of PD-L1, PD-1 is expressed by a variety of activated immune cells, including T cells, B cells, monocytes and dendritic cells. It will be investigated in further study that whether the SCI-induced increase in PD-L1 expression leads more interaction with PD1. At present, the most commonly used animal model for studying Nep after SCI is to evaluate evoked withdrawal behavior, primarily in response to mechanical force and temperature (4). However, the type of pain in patients with SCI is mainly spontaneous and appears without stimulation, with relatively few patients experiencing evoked pains. Therefore, it is increasingly necessary to further evaluate spontaneous ongoing pain after SCI in animal models.

“Time is the spine” has become the central concept of clinical treatment for patients with spinal cord injury. A large number of initially surviving neuronal cells will die within the first few hours after SCI, causing irreversible damage (1). Therefore, quickly diagnosis and early neuroprotective or immunomodulatory interventions have the potential to promote long-term functional recovery and improve the patient’s quality of life. Administration of a high-dose methylprednisolone sodium succinate within 8 hours of injury can significantly improve functional recovery of patients (48). However, there is still a great deal of controversy because of its potential side effects (49). Our findings suggest that early PD-L1 administration can enhance long-term behavioral and sensory function recovery. Given the high potency of PD-L1 in inhibition of inflammatory response and Nep, our findings may facilitate the development of new therapeutic options for SCI.

## Conclusions

Our study elucidated a previously unrecognized function of PD-L1 that underlies promotion of long-term locomotor function and limit mechanical/thermal cutaneous hypersensitivity after SCI. This effect correlated with the promotion of M2-like polarization and inhibition of MAPK signaling pathway.

## Data Availability Statement

The raw data supporting the conclusions of this article will be made available by the authors, without undue reservation.

## Ethics Statement

The animal study was reviewed and approved by Animal Committee of the Naval Medical University.

## Author Contributions

FK, JCS, YW and JGS were responsible for the concept and experimental design. FK, KS, JZ and FDL performed the experiments, data analysis, and statistical analysis. FL, XS, XL, CR and LL provided technical and material support. FK, SZ and KS were involved in drafting and revision of the manuscript. JCS, YW and JGS supervised this study. All authors discussed the results and commented on the manuscript. All authors contributed to the article and approved the submitted version.

## Funding

The study is supported by the National Natural Science Foundation of China, Grant/Award Numbers: No. 81871828, No. 81802218, No. 81702141; Postgraduate Research & Practice Innovation Program of Jiangsu Province (JX10213715).

## Conflict of Interest

The authors declare that the research was conducted in the absence of any commercial or financial relationships that could be construed as a potential conflict of interest.

## Supplementary Material

Supplementary Figure 1 The genotyping of PD-L1 WT or KO mice was confirmed by PCR of DNA samples from tail chips.

Supplementary Figure 2 The overall study protocol.
