# Supplementary figures and images for: PD-L1 Improves Motor Function and Alleviates Neuropathic Pain in Male Mice After Spinal Cord Injury by Inhibiting MAPK Pathway
Source: Front Immunol. 2021 Apr 15;12:670646. doi: 10.3389/fimmu.2021.670646 (PMC8081847; doi:10.3389/fimmu.2021.670646)

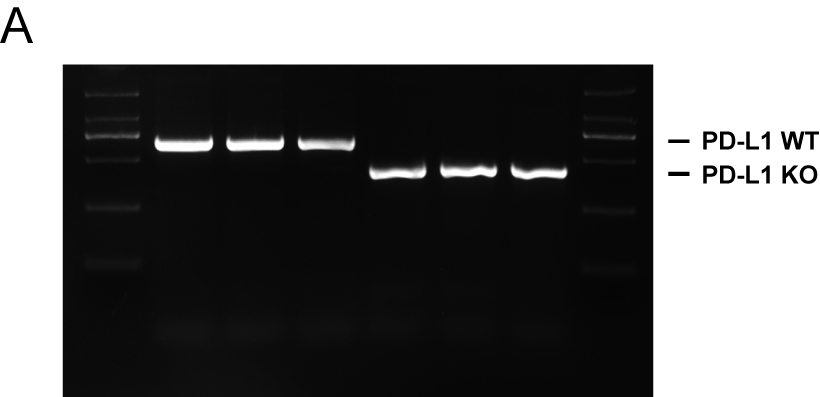

Supplement: Supplementary Figure 1 — The genotyping of PD-L1 WT or KO mice was confirmed by PCR of DNA samples from tail chips. [file Image_1.jpeg]

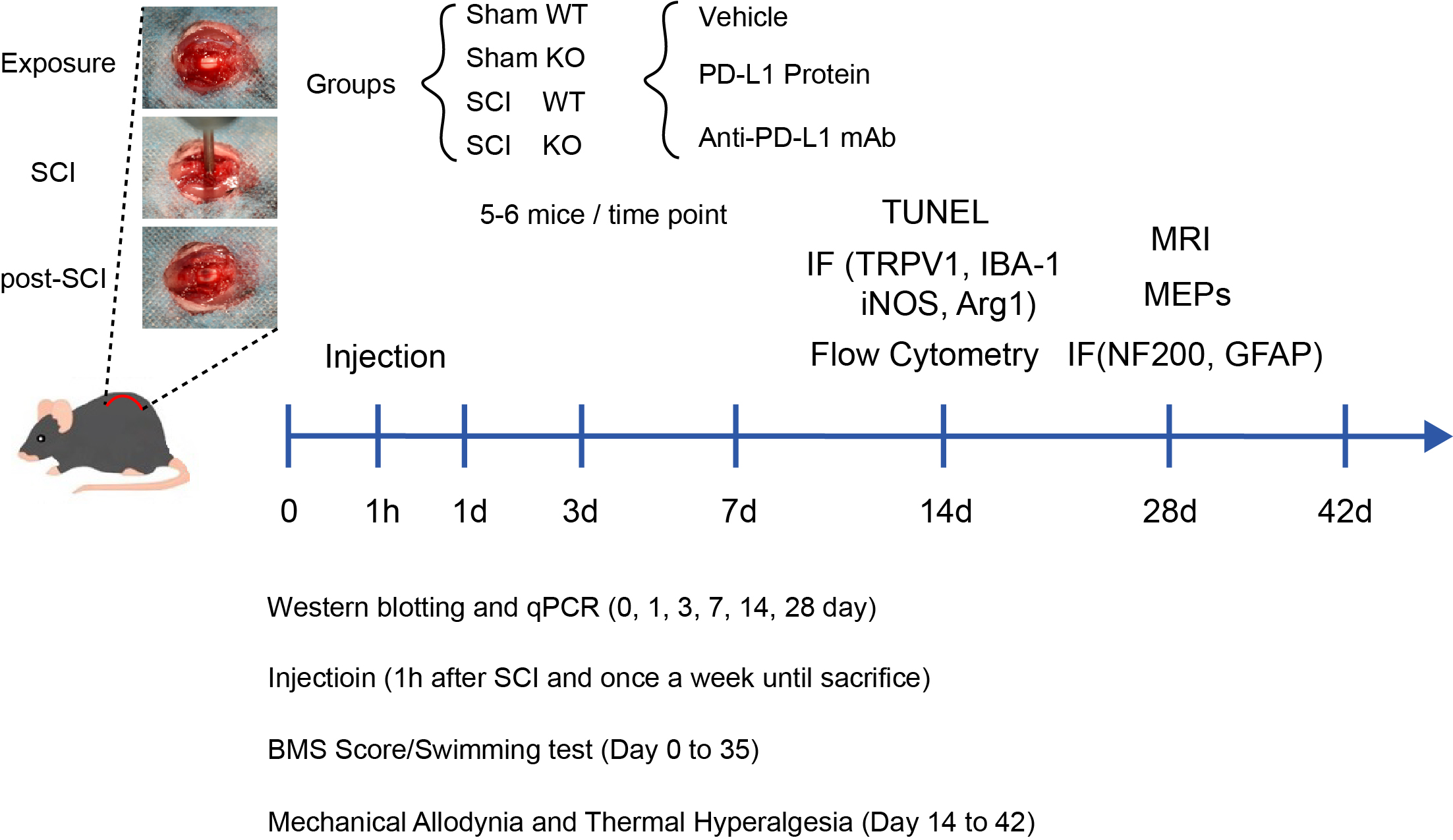

Supplement: Supplementary Figure 2 — The overall study protocol. [file Image_2.jpeg]
